# Primary central nervous system lymphoma in a rheumatoid arthritis patient treated with methotrexate: a case report

**DOI:** 10.1186/s13104-015-1040-0

**Published:** 2015-03-19

**Authors:** Hiromi Shimada, Hiroaki Dobashi, Hisanori Morimoto, Tomohiro Kameda, Kentaro Susaki, Miharu Izumikawa, Yohei Takeuchi, Shusaku Nakashima, Osamu Imataki, Shuji Bandoh

**Affiliations:** Department of Internal Medicine, Division of Hematology, Rheumatology and Respiratory Medicine, Faculty of Medicine, Kagawa University, 1750-1 Ikenobe, Miki-cho, Kita-gun, Kagawa, 761-0793 Japan; Department of Internal Medicine, Mitoyo general hospital, 708 Himehama, Toyohama-cho, Kanonji-si, Kagawa, 769-1695 Japan

**Keywords:** Rheumatoid arthritis, Methotrexate (MTX), Primary central nervous system lymphoma (PCNSL), MTX-associated lymphoproliferative disorders (MTX-LPD)

## Abstract

**Background:**

Rheumatoid arthritis is a systemic inflammatory disease characterized by synovitis and the destruction of articular structures in multiple joints. Methotrexate is recommended as an anchor drug for rheumatoid arthritis treatment to achieve the therapeutic goal of reducing damage to joints and improving clinical score. However, several studies have shown that methotrexate has been associated with the development of lymphoproliferative disorders, namely methotrexate-associated lymphoproliferative disorders. On the other hand, primary central nervous system lymphoma is an aggressive disease with poor prognosis. Both methotrexate-associated lymphoproliferative disorders and primary central nervous system lymphoma are reported to be associated with Epstein-Barr virus.

**Case presentation:**

A Japanese female patient of between 60 and 70 years of age with rheumatoid arthritis was admitted to our hospital because of sudden convulsion and impaired consciousness. Just before admission, she was treated with adalimumab and methotrexate. Contrast-enhanced computed tomography scan showed a densely stained mass with surrounding edema in both frontal lobes and the left nucleus basalis, and enlarged lymph nodes in the right supraclavicular fossa. We performed a biopsy of the right cervical lymph node, but could not establish a histopathological diagnosis. In situ hybridization showed the presence of Epstein Barr virus, therefore we diagnosed this case as methotrexate-associated lymphoproliferative disorders mediated by Epstein Barr virus after considering the drug history of the patient. After we discontinued methotrexate, patient symptoms gradually improved. The masses at both frontal lobes and the left nucleus basalis were gradually regressed.

**Conclusion:**

Since the frequency of methotrexate use and the maximum dosage has been increasing, particular attention should be paid to the development of methotrexate-associated lymphoproliferative disorders in rheumatoid arthritis patients who are treated with methotrexate.

## Background

Rheumatoid arthritis (RA) is a systemic inflammatory disease that is characterized by inflammation of the synovial membrane and extensive, progressive destruction of articular structures in multiple joints. The therapeutic goal of RA is to control such tissue damage and to improve long-term prognosis. Methotrexate (MTX) is the anchor drug for RA treatment, and is expected to suppress the articular destruction. However, several studies have shown that patients with RA have a high risk of onset of lymphoproliferative disorders (LPD) [1], and MTX has been associated with the development of these disorders [2]. In 2008, according to the World Health Organization (WHO) Classification of Tumours of Haematopoietic and Lymphoid Tissues, LPD induced in immunodeficient patients treated with MTX is specifically defined as MTX-associated LPD (MTX-LPD) [3]. Characteristically, MTX-LPD is reported to regress spontaneously following the withdrawal of MTX [4]. Additionally, an association with Epstein-Barr virus (EBV) is indicated [5]. However, primary central nervous system lymphoma (PCNSL) is aggressive and has a poor prognosis [6]. Several reports have indicated that PCNSL is also related to EBV infection, and EBV-related PCNSL occurs more frequently in acquired immunodeficiency syndrome (AIDS) patients and organ transplant recipients with severely decreased immunocompetence [7]. However, EBV-related PCNSL rarely occurs in patients treated with immunosuppressive therapy. Herein, we describe a RA patient treated with MTX who developed MTX-associated and EBV-related PCNSL.

## Case presentation

A Japanese female patient of between 60 and 70 years of age was admitted to the emergency room in our hospital because of sudden convulsion and impaired consciousness in September 2012. She had been diagnosed with RA two decades earlier. She began MTX therapy in 2005, and etanercept was added the next year, but was then changed to adalimumab (ADA) in 2009. Just before admission to our hospital, she was undergoing treatment with ADA (40 mg/2 weeks), MTX (14 mg/week), and predonisolone (PSL) (2 mg/day). In the emergency room, her level of consciousness was E1V1M1 on the Glasgow Coma Scale. Additionally, her upper and lower limbs had a convulsive seizure. Although she did not have anisocoria, her eyeballs were deviated to the left side. After we administered diazepam, her convulsions were improved. However, relapse occurred within a short duration, and the patient was therefore admitted to the intensive care unit.

**Figure 1:**
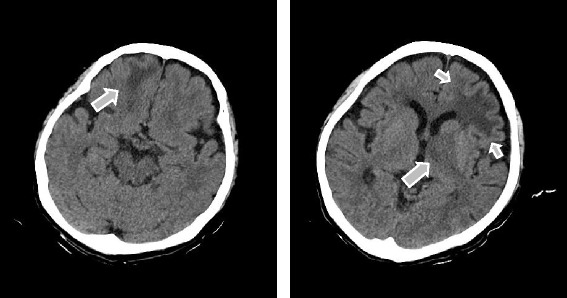
**Cranial computed tomography scan on admission.** Arrows indicated low density areas at the frontal lobes of both sides and the nucleus basalis of left side.

On admission, hematological and biochemical testing (Table 1) revealed an elevated white blood cell count to 14730 per microliter (neutrophils, 64.0%; lymphocytes, 24.0%). C-reactive protein and lactate dehydrogenase were also increased to 0.81 mg/dl (normal range; <0.2 mg/dl) and 306 IU/l (normal range; 110〜220 IU/l), respectively. Immunological test results were within almost normal ranges, except for rheumatoid factor (RF; 64 IU/ml, normal range; <15 U/ml). Procalcitonin and beta-D-glucan were negative, and ferritin and soluble interleukin-2 receptor were within normal ranges. EBV antibody showed a pattern indicative of previous infection (EB-VCA-IgG, 160 titer; EBV-VCA-IgM, negative; anti-EBNA, 80 titer). Cranial computed tomography (CT) scan showed low density areas in the frontal lobes of both sides and the nucleus basalis of the left side (Figure 1). Convulsions disappeared following repeated administration of diazepam. However, we performed artificial ventilation because of severe respiratory depression. Additionally, we discontinued MTX and ADA, and increased PSL dose to 10 mg/day.

**Table 1:** **Laboratory data upon hospital admission**

**CBC**			**Biochemistry**			**Immunology**			**Infection**		
WBC	14730	/μl	CRP	0.81	mg/dl	IgG	1335	mg/dl	PCT	<0.02	ng/ml
Neu.	64	%	TP	7.7	g/dl	IgA	113	mg/dl	β-D	<5.0	pg/ml
									glucan		
Lym.	24	%	Alb	4.4	g/dl	IgM	103	mg/dl	EBV-VCA	0.15	
									IgG		
Mono.	11.5	%	BUN	13	mg/dl	C3	155.9	mg/dl	EBV-VCA	negative	
									IgM		
Eos.	0.5	%	Cr	0.6	mg/dl	C4	27.2	mg/dl	EBNA	0.1	
Baso.	0	%	eGFR	75.99	ml/min.	CH50	63.8	U/ml	T-SPOT Tb	negative	
RBC	391	x104/μl	T-bil	0.7	mg/dl	ANA	1:40, Ho.				
Hb	12.3	g/dl	AST	25	IU/l	RF	64	IU/ml			
Hct	36.8	%	ALT	12	IU/l	MMP-3	33.2	ng/ml	ABG (O2; 3 L)		
Plt	25.8	x104/μl	ALP	246	IU/l	SS-A Ab	10.1	U/ml	pH	7.29	
			γGTP	27	IU/l	SS-B Ab	8.9	U/ml	pCO2	49	Torr
			LDH	306	IU/l	MPO-	<3.0	IU/ml	pO2	74	Torr
						ANCA					
			Na	143	mEq/l	PR3-	<3.5	IU/ml	HCO3	24.3	mmol/l
						ANCA					
			K	4.4	mEq/l	sIL-2R	391	U/ml	BE	−3.4	mmol/l
			Cl	105	mEq/l				SpO2	94.8	%
			Ca	9.3	mEq/l						

White blood cell count was elevated, and C-reactive protein and lactate dehydrogenase were increased.

**Figure 2:**
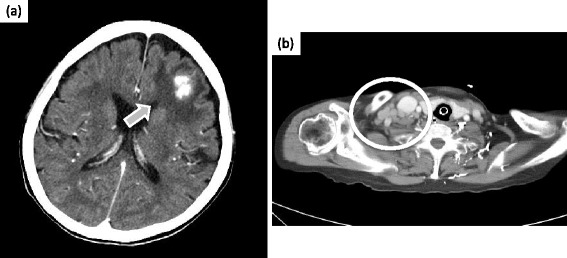
**Contrast-enhanced computed tomography scan on the sixth day after admission. (a)** Arrow indicated showed densely stained masses with surrounding edema at the left frontal lobe, and **(b)** circle showed many swollen lymph nodes in the right supraclavicular fossa.

**Figure 3:**
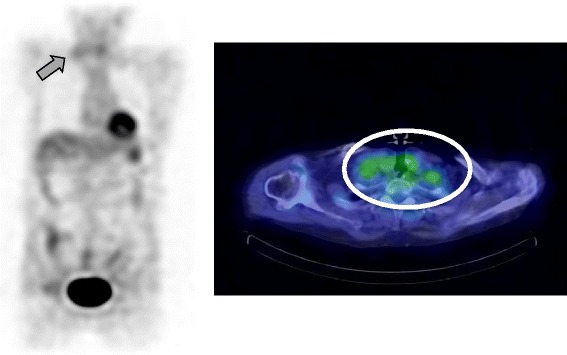
**Fluorodeoxyglucose positron emission tomography/computed tomography scan.** Arrow and circle indicated the accumulation of fluorodeoxyglucose in the swollen lymph nodes of the right supraclavicular fossa.

On the sixth day after admission, we performed a contrast-enhanced CT scan, which showed a densely stained mass with surrounding edema in the left frontal lobes and nucleus basalis, and enlarged lymph nodes in the right supraclavicular fossa (Figure 2). Furthermore, the accumulation in swollen lymph nodes was detected on fluorodeoxyglucose (FDG) positron emission tomography (Figure 3). Therefore, we performed a biopsy of the right cervical lymph node.

**Figure 4:**
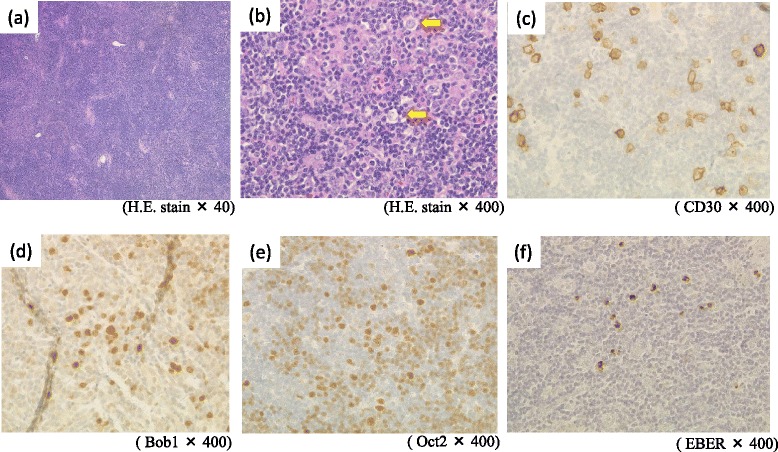
**Biopsy of swollen lymph nodes in the right supraclavicular fossa.** Hematoxylin and eosin staining showed **(a)** the disappearance of follicle structure, and **(b)** dispersed Hodgkin-like large cells with small lymphocytes. **(c)**-**(e)** Immunostaining for CD30, Bob1 and Oct2 showed positive large cells. **(f)** In situ hybridization showed Epstein-Barr virus encoded ribonucleic acid in the nuclei of lymphoma cells.

Histopathological examination of the right cervical lymph node showed the disappearance of follicle structure and dispersed Hodgkin-like large cells with small lymphocytes. These large cells were positive for CD30, Bob1 and Oct2, undeterminable for CD20 and PAX5, and negative for CD15. This finding differed from typical Hodgkin lymphoma, which generally has immunoreactivity for CD30, CD15, and PAX5 (Figure 4 a–e). Many of the small lymphocytes were CD20-positive cells. However, these were negative for MIB-1, and IgH reconstitution was not observed by polymerase chain reaction, therefore we could not diagnose this case as B-cell lymphoma. In situ hybridization showed EBV-encoded ribonucleic acid (EBER) in the nuclei of lymphoma cells (Figure 4f). Consequently, we could not establish a histopathological diagnosis and therefore diagnosed this case as MTX-associated LPD after considering the drug history of the patient.

**Figure 5:**
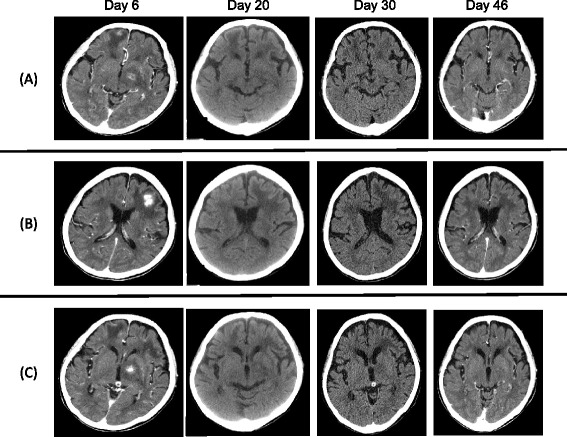
**Cranial computed tomography scan after the withdrawal of methotrexate.** The masses of **(A)** right frontal lobe, **(B)** left frontal lobe, and **(C)** left nucleus basalis were gradually reduced.

After withdrawal of MTX, patient symptoms gradually improved. The masses at both frontal lobes and the left nucleus basalis, and the lymphadenopathy in the right supraclavicular fossa were gradually reduced, as observed by CT scan (Figure 5) and there was no accumulation of FDG after 3 months. The patient was treated with PSL and salazosulfapyridine (1000 mg/day) for RA, and no recurrence of lymphoma was observed more than one year after the discontinuation of MTX.

## Discussion

MTX is an important drug used for the treatment of RA. Hence, over time, the frequency of MTX use and the maximum dosage has been increasing. However, several studies have reported that LPD more readily develops in immunodeficient patients treated with MTX [2]. The characteristics of MTX-LPD are that 1) lymphoma regresses spontaneously following the withdrawal of MTX treatment [4], 2) MTX-LPD is associated with EBV seropositivity [5], and 3) histological findings show various tissue types including diffuse large B-cell lymphoma, Hodgkin lymphoma, polymorphic-LPD, and Hodgkin like-lymphoma [8].

EBV is an oncogenic virus associated with several malignant diseases such as Burkitt’s lymphoma, non-Hodgkin lymphoma, and so on [9]. These malignancies are usually considered to be the result of virus reactivation [10]. EBV was demonstrated in approximately 40% of MTX-LPD cases, and most of them regress spontaneously [3]. The mechanism of MTX-LPD is that the immunosuppressive state induced by MTX treatment may allow reactivation of EBV as normal anti-virus surveillance is impaired, and some MTX-LPDs associated with EBV may regress after MTX withdrawal by the restoration of normal immunity [4].

In our case, a RA patient with a history of MTX administration had a sudden convulsion, multiple brain tumors, and lymphadenopathy observable by CT scan. We could not make a definite diagnosis by histological findings of cervical lymph node biopsy, which demonstrated Hodgkin-like large cells and many CD20-positive lymphocytes. Additionally, EBERs were detected in the nuclei of lymphoma cells. Therefore, we diagnosed MTX-LPD mediated by EBV.

However, we could perform the biopsy not from central nervous system (CNS) lesion but from cervical lymph node. In fact, we could not judge whether the CNS tumor was MTX-LPD or not. After withdrawal of MTX, symptoms gradually improved, and both the CNS tumors and cervical lymphadenopathy remarkably decreased, as observed by cranial CT scan. Hence, we could conclude that these tumors are from same origin.

In this case, ADA was also administered but discontinued after onset of convulsions, concomitantly with MTX. However, Ramiro *et al.* indicated that patients treated with an anti-tumor necrosis factor (TNF) inhibitor did not have an increased risk of lymphoma [11]. Burmester *et al.* showed that the greater rate of lymphoma occurrence observed during ADA trials in patients with RA was within the range that might be expected in a similar RA population not treated with anti-TNF therapy [12]. Therefore, in the present case study, we considered the lymphoma to be caused by MTX and not ADA.

PCNSL occurs in brain, spinal cord, eyeball, cranial nerve, and meninges without affecting other organs. It is aggressive, and almost always of B-cell origin [6]. Although PCNSL is a rare lymphoma that accounts for only approximately 3% of all primary brain tumors, the incidence tends to increase within the last 10 years. High-dose MTX and radiation therapy is recommended as a treatment of PCNSL [13]. Furthermore, previous reports uphold the relationship between EBV and PCNSL [10,14-16]. In general, EBV-related PCNSL has a poor prognosis. Moreover, it occurs in some AIDS patients, organ transplant recipients and a few immunocompetent individuals [7]. Furthermore, there are a few reports that PCNSLs occurred in patients treated with immunosuppressive drugs. Kleinschmidt-DeMasters *et al.* showed four cases of EBV-related PCNSL occurring in older patients who were treated with immunosuppressive drugs (MTX, mycophenolate mofetil, azathioprine, PSL, cyclophosphamide, and so on). In one case, in which the patient was treated with MTX, the lymphoma improved following the cessation of MTX and specific treatment for the toxoplasmosis [17]. Her neurological deficits markedly improved, but she developed renal failure and died on approximately 3 months later. Migita *et al.* also reported a patient with RA who developed an EBV-related PCNSL during treatment with MTX. They underwent surgical resection and MTX withdrawal, resulting in MTX-LPD improvement without any recurrence [18].

In our case, PCNSL markedly regressed only by the discontinuation of MTX, and there is presently no recurrence. This finding confirms the potentiality of the spontaneous nature of MTX-LPD regression following cessation of MTX treatment.

## Conclusion

We have presented a rare case in which a RA patient treated with MTX developed MTX-associated, EBV-related PCNSL, which regressed markedly upon the withdrawal of MTX. Since current treatment strategies for RA recommend MTX as a first choice therapy in early stages of disease, the frequency of MTX use and the maximum dosage has been increasing. Therefore, we need to pay attention to the development of MTX-LPD for RA patients who are treated with MTX.

## Consent

Written informed consent was obtained from the patient for publications of this Case Report and any accompanying images. A copy of the written consent is available for review by the Editor-in-Chief of the journal.

## Abbreviations

RA: Rheumatoid arthritis
MTX: Methotrexate
LPD: Lymphoproliferative disorder
WHO: World Health Organization
MTX-LPD: Methotrexate-related lymphoproliferative disorder
PCNSL: Primary central nerve system lymphoma
AIDS: Acquired immunodeficiency syndrome
EBV: Epstein-Barr virus
ADA: Adalimumab
PSL: Predonisolone
RF: Rheumatoid factor
CT: Computed tomography
FDG: Fluorodeoxyglucose
EBER: Epstein-Barr virus encoded ribonucleic acid
CNS: Central nervous system
TNF: Tumor necrosis factor
